# The Perceived Information in Obtained From the Informed Consent in Iranian Patients With Cancer in Clinical Studies

**DOI:** 10.5539/gjhs.v7n3p1

**Published:** 2014-10-28

**Authors:** Shahrzad Ghiyasvandian, Fariba Bolourchifard, Zohreh Parsa yekta

**Affiliations:** 1 School of Nursing and Midwifery, Tehran University of Medical Sciences, Tehran, Iran; 2 School of Nursing and Midwifery, Shahid Beheshti University of Medical Sciences, Tehran, Iran

**Keywords:** perceived information, informed contest, clinical study, cancer patient

## Abstract

**Objective:**

One of the basic issues in clinical studies is to receive the informed consent; that is to say, all the activities applied in patient’s involvement in the information, decision-making, ability and volunteering in diagnosis, cure and care. In as much as most cancer patients require information about their individual needs, the present study is conducted to determine the perceived information from the informed consent of clinical studies in cancer patients.

**Methods:**

This is a descriptive study. Fifty cancer patients hospitalized for participating in the clinical study was chosen according to the convenience sampling. Tools used in this research included the questionnaire (individual and social features) and the check list about patient’s right and cancer patient’s information before and after receiving informed consent in clinical studies (10 items on a Likert rating scale). To validate the study, content and formal validation was used. Data in this research were analyzed using descriptive statistics (frequency, mean and standard deviation) and the software of SPSS 16.

**Result:**

In general, the mean of the scores obtained from cancer patients’ perceived information before completing the informed consent of the clinical studies was 14±3.5 and after consent of the clinical studies was 16±2.4. The cancer patients’ perceived information before and after consent of the clinical studies was weak.

**Conclusions:**

Based on the findings of the present study, the rate of the information the cancer patients received, before completing the informed consent form, was low, but after completing the informed consent form this rate was again low. Therefore, conducting similar and wider studies is recommended to unveil the factors affecting perceiving information and how to promote the quality of the informed consent in other hospitals in Iran.

## 1. Introduction

A patient’s rights refer to the activities applied to meet the logical and legitimate needs based on the standards, rules and regulations of the medical center members (Kabirzadeh et al. 2009). In Bills of patients’ Rights, the World Health Organization mentioned some items such as having appropriate cure and care, confidentiality of the information, informed consent, independence, education, protest, and redressing (Tahir et al., 2009). The new guideline of patients’ rights creates the right to informed consent, which allows accepting or refusing any medical treatment (Figueroa, 2012). It is a routine practice to get consent before performing procedures in clinical setting (Torrance, 2014; Modra et al., 2014). Informed consent is the process to provide patients with the truthful and essential information in a way which they can understand and recall and permits them voluntarily to make an informed choice on the treatment (Wang et al., 2014).

Clinical research with patient-samples was routinely conducted without informed consent for research participation prior to 1966 (Miller, 2014). Efforts to develop patients’ understanding of their own medical treatments or research in which they are involved are moving ahead, especially with regard to informed consent procedures (Cervo et al., 2013). Although it is generally advised to provide patients with as much information as possible during the informed consent process, little is known about the quantity and type of information that patients really want (Degerliyurt et al., 2010). Researchers must comprehend the applications and requirements of consent inform process. They have legal and moral responsibilities in managing the samples for clinical research purposes to best serve the needs of participants and clinicians and to comply with country laws (Dry et al., 2012).

Clinical trials are carried out with human participants to answer questions about the best way to diagnose, treat and prevent illness (Cresswell & Gilmour, 2014). Clinical trials provide strong evidence but their complex design is difficult for both clinicians and participants to understand. Informed consent is a critical component of clinical research (Synnot et al., 2014). Participants must give informed consent to take part in clinical trials that requires understanding of how clinical trials work and their purpose (Cresswell & Gilmour, 2014). For some treatments, however, there may be disagreement over the requirements for ’informed’ consent (Torrance, 2014).

Different methods of presenting information to potential participants of clinical trials may improve the informed consent process (Synnot et al., 2014). Clinicians and clinician-researchers have important role in the recruitment of patients in the consent process (Kleiderman et al., 2012). Since increasing numbers of patients are asked to take part in clinical trials, nurses need to be aware of the principles of valid informed consent (Pick et al., 2013). The nurses are confronted with a dual role as care provider and researcher. They can apply the moral principles of autonomy, beneficence, and justice to their practice and research related to the informed consent process (Judkins-Cohn et al., 2014).

The literature review showed that in Iran, the information rate of patients with cancer of the informed consent form in clinical researches have not been studied and studies that have been done in this regard, generally focused on the review of patient rights. Cancer is the third cause of death in Iran, after the heart disease and road accidents as the two leading causes of fatality. In addition, Iranians believe that cancer is equivalent to death and end of life (Hamooleh et al., 2013). Based on our experiences in Iranian health organizations the cancer patients complete and sign the informed consent as a routine based bureaucratic process. The cancer patient do not obtain enough information about the elements of inform consent that shows the importance of our study. The purpose of our study that was conducted in research centers of educational hospitals of Tehran Medical Sciences University (in Iran) determines the perceived information in cancer patients about the informed consent of clinical studies.

## 2. Methods

In this descriptive study, the level of the perceived information of the cancer patients about the informed consent of clinical studies was evaluated in research centers of educational hospital of Tehran University of Medical Sciences. After necessary arrangements to obtain the consent of university and related hospitals authorities and visiting the hospitals, the list of the patients in the hospital was provided. When the researchers referred to the chosen samples, 50 patients suffering from cancer who were hospitalized to participate in the study were selected by convenience sampling, and they were explained to accept participation in the study. Tools used in this research included the questionnaire (individual and social features) and the check list (patient’s right and cancer patient’s information before and after receiving informed consent in clinical studies) which were completed by the researcher. In individual and social features, some items such as age, gender, marital status, educational status, job, and type of cure were included.

The checklist had 10 items on a 5 grades Likert rating scale about patient’s right and cancer patient’s information before and after receiving informed consent in clinical studies. The scores were very good (5), good (4), moderate (3), weak (2), and very weak (1). The checklist was categorized in 3 levels include good (38–50), moderate (24–37), and weak (10–23). The total scores ranged from 10 to 50. To determine the validity, content validity and face validity will be used. In so doing, the contents of the checklist were set based on the books and scientific resources, and, based on the information of 5 specialists who have sufficient information about medical ethics and patient’s rights; the contents were modified and completed. Scientific reliability of the test instrument on test retest and observing form was simultaneously applied by researchers, and then the results were compared. Descriptive statistics (frequency, mean and standard deviation) and the software of SPSS 16 were used.

## 3. Results

The individual and social features of the participants are showed in Table 1.

**Table 1:** The individual and social features of cancer patients

Individual and Social features	Number	Percent
Gender		
Male	27	54
Female	23	46
Age		
<30	7	14
30-40	18	36
41-50	22	44
>50	3	6
Marital status		
Single	22	44
Married	28	56
Educational level		
Less than high school diploma	14	28
High school diploma	28	56
More than high school diploma	8	16
Job		
Employed	27	54
Unemployed	23	46
Care and cure		
Surgery	13	26
Chemotherapy	22	44
Radiotherapy	5	10
Adjuvant therapy	10	20

The frequency distribution of cancer patients’ information perception before completing the informed consent of clinical studies concerning each right of the patient is summarized in Table 2.

**Table 2:** Frequency distribution of cancer patients’ perceived information before completing the informed consent of clinical studies based on patients’ right

Patient’s rights in the informed consent of the clinical studies	good	moderate	weak
The purpose of the clinical studies	*28%*	*32%*	*40%*
All risks, side effects or logical expected problems	*20%*	*26%*	*54%*
Any type of logical expected interests	*20%*	*32%*	*48%*
What happens in the study and any procedures, medications, and equipment different from standard are procedure	*20%*	*26%*	*54%*
Other available methods and how better or worse they are compared with the clinical study	*20%*	*32%*	*48%*
To allow asking any type of question both before obtaining informed consent and at any time during the study	*18%*	*34%*	*48%*
To allow making decision to participate in study	*4%*	*26%*	*70%*
Refusing to participate in the study for any reason before or after starting the clinical study	*6%*	*26%*	*68%*
Getting a copy of the informed consent form signed and dated	*16%*	*26%*	*58%*
Any available medical treatment in case of complications during the study	*6%*	*26%*	*68%*

In general, the mean of the scores obtained from cancer patients’ perceived information before completing the informed consent of the clinical studies was 14±3.5. The cancer patients’ perceived information before completing the informed consent of the clinical studies was weak.

The frequency distribution of cancer patients’ information perception after completing the informed consent of clinical studies concerning each right of the patient is summarized in Table 3.

**Table 3:** Frequency distribution of the cancer patient’s perceived information after completing the informed consent of clinical studies concerning patient’s rights

Patient’s rights in the informed consent of the clinical studies	good	moderate	weak
The purpose of the clinical studies	*28%*	*24%*	*48%*
All risks, side effects or logical expected problems	*34%*	*28%*	*38%*
Any type of logical expected interests	*24%*	*34%*	*42%*
What happens in the study and any procedures, medications, and equipment different from standard are procedure	*26%*	*36%*	*38%*
Other available methods and how better or worse they are compared with the clinical study	*28%*	*32%*	*40%*
To allow asking any type of question both before obtaining informed consent and at any time during the study	*15%*	*38%*	*47%*
To allow making decision to participate in study	*14%*	*30%*	*56%*
Refusing to participate in the study for any reason before or after starting the clinical study	*14%*	*30%*	*56%*
Getting a copy of the informed consent form signed and dated	*22%*	*30%*	*48%*
Any available medical treatment in case of complications during the study	*12%*	*16%*	*26%*

In general, the mean of the scores obtained from cancer patients’ perceived information after completing the informed consent of the clinical studies was 16±2.4. The cancer patients’ perceived information after completing the informed consent of the clinical studies was weak.

The findings showed that the information of the cancer patients received, before completing the informed consent form, was low, but after completing the informed consent form this rate was again low.

## 4. Discussion

Based on our study getting informed consent is one of the major parts in clinical studies. Most cancer patients require information about their individual needs and conditions. The purpose of consent is to offer all the activities involved in sharing information, decision -making ability, patient’s volunteer participation in diagnosis, cure and care. Informed consent of trial participants is both an ethical and a legal requirement. The study of Kleiderman et al. (2012) identified general awareness of key ethical issues. They are containing dependence, respect for people, beneficence, non- harmfulness, bias, and privacy that patients contribute to information because of a sense of selfishness and that they want guarantee before consent process for clinical research.

Participants in our study did not know patient’s right about to allow asking any type of question, making decision to participate in study, and refuse to participate in the study for any reason at any time during study. Korotchikova et al. (2010) stated perception of harm was the main reason for declining consent. Researchers must present participants with opportunities to make informed decisions about whether to take part in research studies (Wolbransky et al., 2013; Shiber & Glezerman, 2014). When making a decision about take part in trial, the information provide for samples about the study and have the chance to have any questions answered before their amount of ’informed-ness’ is assessed, usually subjectively, and before they are asked to sign a consent form (Gillies et al., 2014).

Based on our study the perceived information about the purpose of the clinical studies were weak. There are a number of ethical issues related to informed consent, handling and transparency of data in clinical studies (Robbins et al., 2014). Informed consent is the cornerstone of human research subject protection. Many subjects sign consent documents without understanding the study purpose, procedures, risks, benefits, and their rights (Rowbotham et al., 2013). Hammami et al. (2014) indicated the informed consent process is important to patients; however, patients vary in their understandings of its purpose with the dominant view being enabling patients’ self-decision-making. In the other word, most of the consent obtained was not valid because the patients did not understand the purpose of consent (Kabirzadeh et al., 2009).

Our study presented that the cancer patients’ perceived information before and after completing and signing the informed consent form was weak. It seems that is a routine based administrative process of organization. The organization’s aim (such as hospitals and research centers) defines those ethical principles of health care and clinical research. The element of inform consent provides the vital information to make an informed decision regarding health care or participation in clinical research (Abaunza & Romero, 2014). The participants in our study had low information about what happens in the study and any procedures, medications, and equipment different from standard. Sheikhtaheri and Farzandipour (2010) in their study stated that sometimes physicians do not mind getting information to patient. They believe that patients are not capable to understand the information and more information make them misunderstand. Likewise, the study of Hashemi et al. (2010) indicated that nurses and supervisors disagreed to inform the patients about care mistakes which are an option in informed consent. They believes different factor such as lack of patient’s understanding, culture of non-co-operation, impatience and even some patients’ abuse to be involved in their disagreement.

We found in our study the patients’ information about all risks; side effects or logical expected problems were low. On the contrary, the study of Degerliyurt et al. (2010) presented thorough informed consent process generally provides more information than most patients’ desire. Most of patients wanted inform consent provided information before preparation their participation in clinical research and again just before experiencing it (Cervo, 2013). Effective explanation of the medical procedure and the inherent risks and complications, is important determinants of patients’ capacity to provide fully informed consent (Sherlock & Brownie, 2014). In this regard, most of the participants in our study did not obtain any information about any available medical treatment in case of complications during the study. They did not have a copy of the informed consent form signed and dated. Some patients were of the opinion that the primary purpose of the consent procedure was to protect hospitals and researchers. They considered the consent as clinicians and researcher’s protection against legal issues (Tahir et al., 2009).

Also, the patients’ perceptive of the medical procedure, risks and complications is often low (Sherlock & Brownie, 2014). The results of our study are consistent with the result of a study conducted by Peyrovi et al. (2013) in which it is said all patients have little information about their illness, treatment and complication. In addition, Afolabi et al. (2014) described that the comprehension of key concepts of informed consent is poor among study participants across Africa. The finding of these researches may be explained that patients do not like to obtain information in this regard and on the other hand, researchers did not provide the necessary information for samples.

According to our research the cancer patients’ perceived information about the purpose of the clinical studies, after completing the informed consent was better than before but it was weak yet. Based on our clinical and educational experience, the potential reason may be the requirement of educational program for researchers and research participants about informed consent to facilitate its process in Iran. If the patients get enough information about health care and clinical research they will be more satisfied and less complaint. Therefore, conducting similar and wider studies is suggested to identify factors involved in perceiving information and promoting the quality of getting informed consents in Iran’s other hospitals.

## 5. Conclusion

In this study, the patients’ right to informed consent in clinical studies was scrutinized. The findings of this research show that there is weak degree of patients’ understanding and awareness with the informed consent in clinical studies. The results of our study indicated that a completed and signed inform consent form does not mean that cancer patient’s perceived information about clinical studies. Although the understanding of cancer patients before and after completing the informed consent of clinical studies was low. The findings of our study could be an appropriate criterion for qualitative and qualitative elaboration for getting informed consent in clinical research as well as clinical practice.
